# The uses and abuses of tree thinking in cultural evolution

**DOI:** 10.1098/rstb.2020.0056

**Published:** 2021-07-05

**Authors:** Cara L. Evans, Simon J. Greenhill, Joseph Watts, Johann-Mattis List, Carlos A. Botero, Russell D. Gray, Kathryn R. Kirby

**Affiliations:** ^1^ Department of Linguistic and Cultural Evolution, Max Planck Institute for the Science of Human History, Jena 07745, Germany; ^2^ ARC Centre of Excellence for the Dynamics of Language, ANU College of Asia and the Pacific, Australian National University, Canberra 2700, Australia; ^3^ Religion Programme, University of Otago, Dunedin 9016, New Zealand; ^4^ Centre for Research on Evolution, Belief and Behaviour, University of Otago, Dunedin 9016, New Zealand; ^5^ Department of Biology, Washington University in St Louis, St Louis, MO 63130, USA; ^6^ School of Psychology, University of Auckland, Auckland 1010, New Zealand; ^7^ Department of Ecology and Evolutionary Biology, University of Toronto, Toronto, ON, Canada M5S 3B2

**Keywords:** cultural evolution, phylogenetic comparative methods, cross-cultural research, cultural macro-evolution

## Abstract

Modern phylogenetic methods are increasingly being used to address questions about macro-level patterns in cultural evolution. These methods can illuminate the unobservable histories of cultural traits and identify the evolutionary drivers of trait change over time, but their application is not without pitfalls. Here, we outline the current scope of research in cultural tree thinking, highlighting a toolkit of best practices to navigate and avoid the pitfalls and ‘abuses' associated with their application. We emphasize two principles that support the appropriate application of phylogenetic methodologies in cross-cultural research: researchers should (1) draw on multiple lines of evidence when deciding if and which types of phylogenetic methods and models are suitable for their cross-cultural data, and (2) carefully consider how different cultural traits might have different evolutionary histories across space and time. When used appropriately phylogenetic methods can provide powerful insights into the processes of evolutionary change that have shaped the broad patterns of human history.

This article is part of the theme issue ‘Foundations of cultural evolution'.

## 1. Introduction

Theories of cultural evolution are built on the observation that cultural features undergo innovation, modification and transmission. Over time, these processes have generated remarkable variation in human cultures. Humans speak around 7000 distinct languages, affiliate with hundreds of religions, employ a range of kinship systems, engage in an array of subsistence practices and adhere to a bewildering number of social conventions [1]. Phylogenetic methods provide a powerful approach to studying macro-evolutionary patterns of innovation, modification and transmission [2–4]. Their application to human culture has helped reinvigorate cross-cultural comparative research but has also been subject to criticism—both valid and misguided.

**Figure 1.**
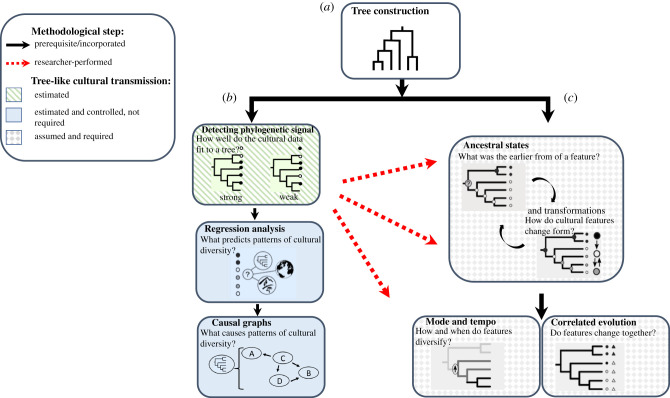
Phylogenetic methods that can be used to study cultural macro-evolution. Black arrows indicate that the preceding methodological steps are directly incorporated in later methods: (*a*) tree construction [5] is required for all subsequent steps; (*b*) testing for phylogenetic signal (e.g. [6–8]) forms an integral part of phylogenetic regression (e.g. [9–11]), which in turn forms the basis of phylogenetic path analysis which can identify causal relationships; (*c*) ancestral state reconstruction (e.g. [12]), estimated in conjunction with rates of trait change and transformation (e.g. [13,14]), is required for models of trait correlation [15–17] and diversification ([18,19]; but see [20]). Red arrows indicate that suitable tests of phylogenetic signal (i.e. that the trait data fit sufficiently to the history inferred by the tree) should be conducted by the researcher before using methods detailed in (*c*); (see also §2). Shading: grey shading indicates methods that both assume and require inferred historical relationships between the cultural units (tree taxa) to sufficiently reflect the history of the trait; green shading denotes methods that detect and quantify tree-like structure in cross-cultural data; blue shading denotes methods that detect and control for tree-like data structure among societies, but do not require it.

Phylogenies, also known as evolutionary trees, represent the common ancestry of populations and the splitting events that have occurred over the course of their history. Phylogenetic methods encompass a broad family of mathematical approaches that can be used to construct, analyse and incorporate phylogenies (figure 1). Originally developed to study the evolution of biological organisms, these methods offer a general toolkit with the potential to provide answers to a range of cultural evolutionary questions.

An important distinction in cultural phylogenetics research is between methods of building trees (i.e. reconstructing the histories of cultural units based on assumptions of vertical transmission of cultural features (traits); figure 1*a*) and methods that use previously constructed trees in models that investigate the evolution and distribution of other cultural traits (figure 1*b*-*c*). A further important division in tree thinking occurs between those methods and questions that simply detect and control for tree-like structure when examining variation in cross-cultural data (e.g. *What does the distribution of traits among societies tell us about the history of those societies and/or traits? Does horizontal or vertical transmission better explain the observed distribution of traits?* figure 1*b*), and those methods that require that the modelled data *are* tree-like (i.e. methods that ask: *What was the ancestral form of a cultural feature?* figure 1*c*).

Phylogenetic methods offer exciting possibilities for a wide range of questions, only some of which explicitly require tree-like data. For data that *are* sufficiently tree-like, one of the strongest appeals of phylogenetic methods is that they offer the possibility to illuminate the unobservable past. Phylogenetic methods can reconstruct the ancestry of a vertically transmitted trait from the evolutionary signatures detected in its present-day distribution, even when archaeological records are entirely unavailable. However, despite this exciting potential, debate continues over how best to integrate cultural heterogeneity, disentangle the signatures of vertical transmission, horizontal diffusion and local socio-ecological drivers, and demonstrate that a cultural trait exhibits *enough* tree-like structure to justify using methods that reconstruct its evolutionary past.

Here, we review the application of phylogenetic methods in cross-cultural research. We focus specifically on the questions researchers should ask in order to avoid common methodological pitfalls when (i) deciding about the units of the underlying cultural data, (ii) constructing trees and (iii) assuming tree-like transmission of other cultural features. Throughout, we outline a series of best practices and highlight emerging methods that promise to advance our understanding of macro-evolutionary patterns of mechanism and causation in culture.

## 2. Are the data appropriate for comparative phylogenetic analysis?

In the social sciences, phylogenetic methods have been used to build trees representing the evolution of a broad range of cultural units, including manuscripts [21], stone projectile points [22], textiles [23], languages [18,24–27], social systems [28] and nation states [29]. Cultural units—also referred to as taxa—are the entities represented at the tips of phylogenetic trees. A given cultural unit—or taxon—is typically compared with another based on its attributes (features or traits). Below we outline three key considerations related to the structure, type and quality of the data used to delineate and describe cultural units and their traits. If ignored, the validity and reliability of inferences that can be drawn from the phylogenetic analyses described in figure 1—i.e. methods that construct trees and methods that use previously constructed trees in models that investigate the evolution and distribution of other cultural features or traits—may be undermined by the data.

First, trait data should be structured around comparable units of analysis. This helps ensure variation in trait data reflects differences in the units' evolutionary histories, rather than differences in the scale of units being studied. For instance, the Database of Religious History contains descriptions of units varying in scale from single-church communities, through religious families, to political empires [30]. The evolutionary histories of the units, and the evolutionary processes acting on the units, are expected to vary across these different scales. Thus, researchers seeking to use phylogenetic methods to investigate these data should ensure their sample consists of religious units of a similar scale, and also that there is compatibility between the scale of the religious units and the units represented by the tree (see also [31,32]).

Second, ‘traits' must also represent comparable ‘entities' across taxa [33]. For example, cognate coding of word lists assumes concepts represented in the lists (e.g. bird, hand) were defined the same way across languages. Similarly, meaningful comparison of artefact morphology (e.g. projectile traits) requires that measurements across taxa be based on consistently identifiable measurement start- and endpoints. In the case of cultural behaviours or practices, a trait (e.g. moralizing high gods) must be defined in such a way as to be identifiable in very different contexts. Close attention to the ways traits (concepts, morphological measurements and cultural practices) were defined for different taxa (languages, artefacts and societies) is critical to the quality of subsequent analyses.

These concerns are increasingly being addressed through the design of transparent and dynamic cross-linguistic and cross-cultural databases [32]. This includes making available and linking detailed metadata to published datasets (e.g. detailed trait definitions, coders' notes on uncertainty and links to primary sources), so that the definitions used when coding ‘traits' are clear [1,34]. However, even complete with their metadata, coded datasets like Murdock's [35] Ethnographic Atlas are often limiting in that they delineate traits and alternative trait states (codes) based on the research interests and theories of the era in which they were built. In addition, they contain relatively little documentation on how coding decisions were reached, making it hard to evaluate the validity of the data [36]. An alternative model is provided by the eHRAF (Human Relation Area Files) World Cultures [37], which provides users with finely indexed, searchable primary ethnographic materials.

Finally, variation—or conflict—of a given trait within a cultural unit, and variation in the focal dates of cross-cultural observations must be considered when selecting data for phylogenetic analysis [38]. Variation in the expression of traits within a cultural unit is not commonly represented in cross-cultural datasets despite the potential to be widespread for many traits. For instance, societies can be assigned up to two different codes for a number of variables in the Ethnographic Atlas [35], including ‘exchanges at marriage', ‘post-marital residence location' and ‘house shape'. Meanwhile, conflicting reports of the expression of a trait for a taxon are not uncommon and are often reported in coding notes (e.g. [35]), or may be represented in multiple, conflicting entries by different historical experts [30]. Additionally, the difficulty of obtaining synchronous data for multiple taxa can result in trait data for different taxa being based on observations collected over a span of several decades/centuries [39].

Such heterogeneities, contentions and inconsistencies within the data sample can, of course, interfere with the accuracy of the inferences obtained from phylogenetic methods, which often assume single, unambiguous trait values at the tree tips for a given taxon, and sometimes require that trait measurements are synchronous in time (i.e. require, among other things, ultrametric trees). That said, an increasing number of comparative cultural studies are focusing on measures of variance (e.g. [40]) or measures of elasticities (or functions or associations; e.g. [41]). In phylogenetic analyses, an increasing number of solutions are being offered by Bayesian approaches that allow intra-taxon variation [11] and ambiguity in the expression of traits [42] to be incorporated in analyses (for more information on why Bayesian methods are preferred, and further information on choosing models and priors, see [43–45]). In addition to considering emerging methods for accounting for intra-taxon variation, we encourage researchers to consider the sensitivity of their inferences to both trait-measure inaccuracies and interference resulting from trait lability across varying sampling time windows (see [46] for an example). Together, these practices will improve the quality and reliability of results obtained from the application of phylogenetic methods to a given cultural dataset.

## 3. Tree construction: are phylogenetic trees accurate representations of cultural histories?

Phylogenetic methods have the potential to influence our understanding of the evolution of specific domains of culture (languages, artefacts) as well as of the histories of the populations (or other cultural units) with which these cultural domains are associated. Phylogenetic methods can also provide a framework for cultural transmission against which alternative hypotheses about cultural change can be tested. Given this potential, how can we be sure that the trees resulting from phylogenetic analyses are accurate representations of cultural histories? In the sections below, we outline a two-pronged approach to validate cultural phylogenies—combining simulation studies and careful benchmarking (e.g. [46,48]). We also emphasize the importance of using probability-based methods that estimate uncertainty in inferences about the tree topology.

### (a) Simulating trees

The first validation of tree construction methods is whether they can recover known trees after ‘laboratory' manipulations have introduced ‘noise' into the underlying data. A major concern with using trees as representations of history is that cultures can transmit information horizontally between groups through processes like cultural diffusion or linguistic borrowing [49,50]. Phylogenetic methods might be expected to break—i.e. give the wrong result—if there were traits in the underlying data that had been horizontally transmitted rather than inherited vertically from parent to daughter lineages. To test this, Greenhill *et al*. [51] constructed a simulation study where they took two known phylogenies and used these trees to simulate datasets under varying levels of horizontal transmission, ranging from none up to a very high rate of 50% of all traits in the data being borrowed every 1000 years. Then for each of these simulated datasets, they used Bayesian phylogenetic methods to reconstruct the phylogeny and compared the reconstructed phylogeny with the original known phylogenies. These results showed that Bayesian phylogenetic methods were, in fact, highly robust to borrowing—able to correctly recover trees very similar to the original ones even under quite high levels of borrowing of approximately 15% every 1000 years.

However, this does not mean that borrowing is not a problem. Greenhill *et al*. [51] also showed that the effect of borrowing is larger on unbalanced tree topologies with shorter branches, and when borrowing is concentrated between a small set of branches. Further, studies investigating the effect of horizontal transmission on trait mapping have been less positive, with Nunn *et al*. [52] arguing that their simulation results show that phylogenetic comparative methods should only be used when vertical transmission of traits is almost certain. However, a response by Currie *et al*. [14] argues that the rates of horizontal trait transmission simulated by Nunn *et al*. were unrealistically high, and in fact the results from the phylogenetic comparative methods did not perform any worse than linear regression. Our recommendation is that caution is still needed when high rates of borrowing are to be expected, and that the effects of horizontal transmission are likely to interact with the tree shape and trait rates. Of course, with much higher levels of borrowing, the initial pattern of vertical descent can become obscured. For example, in a phylogeny of football [48], geography trumped genealogy. Canadian football appeared closer to varieties of American football despite being historically derived from Rugby Union. The challenges of mechanistic (un)identifiability are discussed in more detail in §4.

### (b) Benchmarking trees

Once constructed, trees should be benchmarked against other representations of the evolutionary history derived from alternative methods, data and approaches. Studies on a wide range of language families have generally found high concordance between the subgroupings identified by the traditional linguistic comparative method and Bayesian phylogenetic analysis of basic vocabulary: e.g. [26] (Sino-Tibetan), [27] (Dravidian) and [24] (Bantu). For example, [18] inferred the phylogeny of 400 Austronesian languages spoken in island Southeast Asia and the Pacific. To validate their result, they compared the subgroupings inferred on their trees with those proposed by traditional linguistic methods and found a very high concordance (e.g. [53]). Of the 400 languages, only 25 could be considered to be possibly misplaced, and, critically, none of these misplacements disrupted the overall shape and pattern of the remaining languages ([54,55]; though see also [56]). Furthermore, many of the misplaced languages are involved in ongoing debates about their correct placement, indicating that the phylogeny is reflecting fundamental existing problems with the placement of these languages [54]. Most strikingly, the Austronesian basic vocabulary trees showed a close fit with both the expansion sequence and timing inferred from both archaeological and genetic data [57,58]. All three types of data support an initial origin in Taiwan approximately 5000 years ago, followed by a series of expansion pulses and pauses across island Southeast Asia and the Pacific. The basic vocabulary tree reflects this initial expansion across the Pacific even though there was subsequently a very substantial influx of people with ‘Papuan' genomes in regions such as Vanuatu [59].

### (c) Quantifying uncertainty in the tree

Finally, the advent of Bayesian phylogenetic inference offers researchers the advantage of constructing a posterior distribution of possible phylogenies, whereby each unique tree topology and set of parameter settings is represented by its posterior probability. Bayesian inference sits in contrast with previous methods of phylogenetic reconstruction—which instead produce point estimates of the single best tree—allowing researchers to consider both the range of theoretically possible trees and the degree of ‘certainty' with which the tree topology can be estimated [60–62]. Moreover, many downstream methods that use the tree to investigate the evolution of other traits (figure 1*b,c*) can use the posterior distribution of trees to integrate their inferences over uncertainty in the tree estimate.

## 4. Mapping other cultural features to lexical trees: divergent evolutionary histories, mechanistic (un)identifiability and model shortcomings

We propose that the cultural trees that best match population history are generally those constructed from the basic vocabulary. They typically exhibit relatively high levels of tree-like vertical transmission, show robustness to realistic levels of borrowing, and match the archaeological and historical records (see above). Once reliable linguistic trees are constructed (figure 1*a*), they can be used to reconstruct the evolution of other cultural features (figure 1*c*), to test and control for the phylogenetic non-independence of cultural units and to examine alternative hypotheses about the forces underpinning phylogeographic distributions of particular cultural features (figure 1*b*). However, in each of these cases, there are potential pitfalls that should be avoided and which we explore in the following subsections.

### (a) Do different cultural traits have different evolutionary histories?

The extent to which basic vocabulary trees should be assumed to be good evolutionary models for other types of linguistic features (e.g. wider components of the lexicon or typological features) or for non-linguistic cultural traits (e.g. norms, rituals, subsistence and social structure) remains a topic of debate and a subject of empirical inquiry [50]. The potential for trait-to-tree mismatch causes more serious methodological concerns for approaches that require the cultural data to map to the same history as the tree (figure 1*c*) than methods that estimate and control for tree-like non-independence between cultural units (figure 1*b*).

Boyd *et al*. [63] provide a useful framework for considering whether a given cultural trait, or set of traits, is likely to mirror the evolutionary history represented by basic vocabulary trees. At one extreme, Boyd *et al*. envision cultures as loose collections of ephemeral entities, dominated by horizontal transmission, and without integrated vertical transmission. At another, cultures are described as discrete entities that evolve as tightly integrated systems, as with vertebrate species (but not bacteria and viruses). A third possibility might be that cultures consist of core, vertically inherited traditions, but also contain some peripheral traits that are subject to much greater horizontal transmission. Finally, cultures could represent assemblages of coherent clusters, whereby phylogenetic methods might be appropriate on a cluster-by-cluster basis, but only if cluster boundaries and the relative importance of vertical versus horizontal transmission within clusters can be identified. Related to this last scenario is the phenomenon of incomplete lineage sorting, well known in evolutionary biology as a source of historical incongruences.

In biology, incomplete lineage assortment occurs when differences in genealogies are observed across some genetic loci and also with the overarching species tree. It results from incomplete sorting of an ancestral polymorphism during successive speciation events, leading to gene trees that coalesce at different points in time [64]. Remarkably, for instance, whole-genome analysis of the great apes has indicated that approximately 30% of our genome supports that humans split from chimpanzees earlier than they split from gorillas, or that these two species split from humans earlier than they split from each other [65]. That gene trees are not always congruent with species trees highlights the inevitability that cultural trait phylogenies will not always display the same topology even when the underlying cultural history is completely tree-like.

Incomplete lineage sorting might be quite common in language evolution, given the multiple sources of polymorphism that characterize language change [66]. There are two major sources for polymorphisms in language evolution—sociolinguistic variation and linguistic variation. Neither of these two sources of variation can be easily filtered out during data preparation, which is why it is important to consider both when using linguistic data to make evolutionary inferences. Sociolinguistic variation refers to the fact that social factors can generate individual differences in language use, preferences, and even grammars between speakers of the same language [67]. Linguistic variation refers to variation in language traits arising through complex processes of differential transmission. For example, many linguistic traits represent a complex of smaller entities that tend to evolve together; as a result, languages may exhibit certain parallel changes long after separation, a phenomenon commonly known as drift among linguists [68,69]. The resulting evolutionary scenarios can be difficult to reconcile with a single tree.

**Figure 2.**
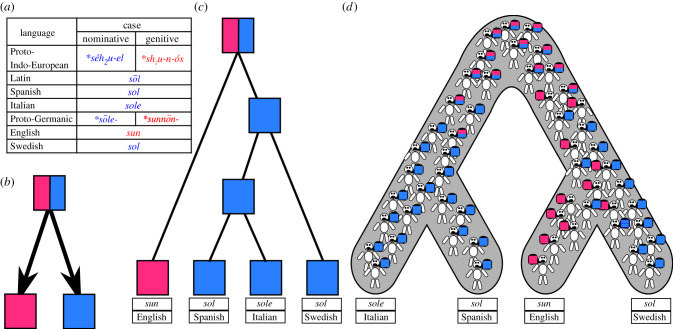
Incomplete lineage sorting in language evolution. The table in (*a*) shows words for ‘sun’ in Indo-European languages along with intermediate stages, with two different forms for nominative and genitive case in Indo-European (similar to the irregular plural of mouse/mice in English), reflecting linguistic variation, and two independent word forms postulated for Proto-Germanic, reflecting sociolinguistic variation; (*b*) shows the basic linguistic change scenario by which either the nominative or the genitive form is preferred as a base form in complex paradigms; (*c*) shows a parsimonious but wrong language tree for extant languages, and (*d*) shows a reconciled scenario.

Figure 2 expands on List [70] with an example informed by Kroonen [71] to illustrate how the seemingly non-tree-like evolution of linguistic traits can be reconciled with an overall language phylogeny by invoking both sociolinguistic and linguistic variation. Here, linguistic variation is reflected in the fact that words in the Indo-European languages often have complex paradigms that were differently transmitted during the evolution of the Indo-European languages. As a result, a word like English ‘sun' reflects an ancient genitive (or more properly ‘oblique’) form, while a word like Spanish ‘sol’ reflects an ancient nominative (rectus) form. Although both languages are related, they show different word forms for the concept ‘sun', owing to a specific form of linguistic variation that was inherited by many descendants of the Indo-European language. But linguistic variation is only one factor contributing to patterns that seem to contradict a given phylogeny. Another very common source of variation is sociolinguistic variation due to the fact that each language variety is spoken by many speakers at the same time who may well pronounce words differently or prefer certain words over other ones, in different contexts. According to Kroonen [71], this must have been the case in Proto-Germanic, the ancestor language of Swedish and English, where two words for ‘sun' seem to have been in use at the same time, one that later became the ancestor of English *sun*, and one that later became the ancestor of Swedish *sol*. While the English form ultimately goes back to the old genitive and the Swedish form goes back to the old nominative paradigm form, the intermediate stage that can be reconstructed for Proto-Germanic was not a stage of one word showing two different case forms, but rather of two words that we assume were used in different contexts or preferred by different speakers in the same language community.

Importantly, using phylogenetic methods does not entail a commitment to the assumption that there is only one history. Indeed, phylogenetic methods can be used to investigate the extent to which different aspects of culture have congruent histories, and even to test alternative explanations for apparent incongruencies. For example, Matthews *et al*. [72] formally tested whether the motifs in pile-weave and non-pile-weave Iranian textiles shared the same history or not. They found that the two traditions were best explained by different phylogenies. Another study by Greenhill *et al*. [73] mapped lexical and grammatical data onto a phylogeny of Austronesian languages. So as to not bias the results in favour of the lexicon or grammar, the phylogeny was derived from independent historical linguistics research with subgroupings often primarily defined by phonological innovations, rather than grammatical or lexical features. They found stark differences in how these two subsystems of language tracked the phylogeny: they were evolving at different rates (on average, the lexicon changed slower than the grammatical data), and the grammatical data showed a poorer fit to the phylogeny, with more conflicting signal (homoplasy), probably caused by higher rates of horizontal diffusion. Greenhill *et al*. argue that these different subsystems of languages have differing dynamics that need to be carefully teased apart. Teasing these patterns apart is often only possible once you have a good estimate of the phylogeny. However, a recent study by Verkerk [74] has applied a new ‘multiple topologies' method [75] that simultaneously infers tree topology while assigning characters to these topologies. Methods like those in Matthews *et al.* [72], Greenhill *et al*. [73] and Verkerk [74] provide a promising way forward for investigating the congruence of cultural histories without forcing all traits to share the same history.

### (b) When is the use of methods that require historical coherence justified?

One subset of phylogenetic methods *does* require coherence and vertical transmission of cultural features (figure 1*c*). Identifying *a priori* which features can be used in phylogenetic reconstructions is often challenging, because of interference from processes such as horizontal transmission, incomplete lineage sorting and independent invention (e.g. convergent evolution in ecologically similar environments). The debate over how best to demonstrate that a cultural trait exhibits *enough* tree-like structure to justify using methods that reconstruct its evolutionary past (figure 1*c*) continues. We believe that the key to progressing against these difficulties is a three-step approach that considers (1) the mode of transmission, (2) benchmarking practices against alternative lines of evidence and (3) the continued development and careful utilization of (i) methodological advances that disentangle pattern from process and (ii) alternative analytical approaches that can be applied when the tree-like structure is missing or questionable in the data. Further discussion of the relationship between the histories of language phylogenies and cultural traits is provided in Slingerland *et al.* [32].

#### (i) Modes of transmission and expression

First, it can be useful to consider what is already known about a trait's mode of transmission within and between groups, the mode of expression (individual versus group) and the extent to which a focal trait's expression and transmission can be disentangled from that of other traits [46,76]. For instance, the present-day global distribution of a cuisine like pizza, which came into being in late eighteenth-century Naples, reveals much about the history of migration and economics, and relatively little about the cultural inheritance of food preferences. Similarly, the spread of major world religions might show relatively tree-like structure in their nested pattern of schisms, but any resulting trees of religion that could be generated from this nested structure would represent much more recent historical events than trees based on basic vocabulary. Attempting to reconstruct the history of Christianity on an Indo-European language tree would thus make no sense.

#### (ii) Benchmarking and validation

Currie *et al*. [14] used phylogenetic comparative methods to infer the evolution of political complexity on the Austronesian tree and provided a good example of trait-history benchmarking and validation. Their results showed that, in the Pacific, political complexity generally increased sequentially in small steps but that decreases in complexity could also happen through bigger drops (i.e. ‘collapses'). Crucially, they were able to validate these inferences by comparing them with the historical, linguistic and archaeological records. For example, the ancestral societal organization of Proto-Oceanic was phylogenetically inferred to be limited to local communities, which is consistent with archaeological evidence showing only small-scale settlements [77]. Furthermore, archaeological and linguistic evidence also indicates that it is likely that this society had some form of social ranking [78], which later became elaborated into simple chiefdoms such as those in the Trobriand Islands and then further elaborated into complex chiefdoms in Polynesia like those in Tonga and Hawaii [14,79].

In another paper using the same language tree, Sheehan *et al*. [16] investigated the coevolutionary relationship between intensive resource use and sociopolitical hierarchy, finding support for a reciprocal relationship between these two variables and highlighting the importance of both social and material factors as drivers of cultural complexity. Here, the authors were able to validate their phylogenetic trait reconstructions with evidence from the archaeological record: models that were constrained by the known history of intensive resource use were consistent with, and provided validation for, models that were given no constraints. In yet further analyses from the same region, [17] highlighted that the relatively recent emergence of moralizing high gods, as indicated by their ancestral state reconstructions, is consistent with early Muslim trade patterns in Southeast Asian cultures, and that the concept of moralizing high gods was ‘borrowed in' to these societies during contact with traders. All the examples illustrate the fundamental role that historical benchmarking can play in validating cultural phylogenetic inferences, as well as the value of phylogenetic methods in contexts where horizontal transmission is important.

### (c) What about methods that detect yet do not require tree-like structure in the data?

Of course, many cultural traits do not leave any traces of their histories in the ‘fossil' record. Methods that detect and quantify the strength of the tree-like structure in the data offer another line of validation and do not assume tree-like evolution *a priori*. In fact, all traits being considered for phylogenetic reconstruction should first be formally examined for phylogenetic signal against the proposed tree model (see e.g. [15]). When a lack of tree-like signal is detected, and/or considerations of transmission mode or benchmarking imply that a trait is unsuitable for methods involving phylogenetic reconstruction (i.e. figure 1*c*), phylogenetic methods that do not require tree-like data—but instead model the contemporaneous distribution of cross-cultural variation while controlling for detectable phylogenetic signal—can offer informative alternatives (i.e. figure 1*b*). These latter methods might even be preferred, when a number of different traits and variables are being modelled, as phylogenetic reconstructions limit researchers to investigations of a maximum of two binary traits in coevolutionary models. Minocher *et al*. [10], for example, using phylogenetic regression, found that marriage patterns in a globally representative sample were best explained by pathogen-stress and male intra-sexual competition, after including eleven predictor variables, spanning a range of potential socio-ecological hypotheses, in their model. Exciting recent developments build further on this regression approach, allowing cross-cultural analyses to test hypotheses concerning directional causality in contemporaneous trait distributions—i.e. methods that detect shorter-term causal changes and do not require estimations of ancestral evolutionary states or processes—using phylogenetic path analysis [80].

In the next two sections (§§4d,e), we will discuss how tests of the phylogenetic signal can also be problematic as they can falsely appear to have high phylogenetic signal when alternative evolutionary processes—such as horizontal transmission, or independent invention in spatially or ecologically correlated environments—are not appropriately modelled and controlled.

### (d) Are there correlations between the drivers of cross-cultural similarity that create a false impression of ‘fit' to the language tree?

The extent to which phylogenetic methods can partition the different evolutionary drivers that have contributed to the present-day phylogeographic distribution of a trait remains controversial. This involves teasing apart the relative importance of convergent evolution, horizontal transmission and vertical transmission. One of the greatest challenges for making inferences on the evolution of culture is that very different sources of cultural similarity can lead to virtually identical phylogenetic and geographic trait distributions in the present. For example, a trait may be spatially clustered because it is adaptive under a specific set of environmental conditions (habitat types are often spatially clustered themselves), because it was jointly inherited by a group of neighbouring societies that descend from a common ancestor, or because it was more likely to diffuse horizontally among nearby groups that interact frequently with each other. Because neighbouring groups are also typically close relatives and tend to inhabit similar habitats, distinguishing the effects of these correlated mechanisms can be extremely challenging. The continued development of methodological advances offers the most likely promise of resolution to this particular issue.

#### (i) Methods to decouple ancestry, space and ecology

Traditionally, cultural evolutionists have adjusted their sampling regimes to break down, at least in part, the correlation between diffusion, vertical transmission and environmental selection in cross-cultural studies, hence removing the requirement to quantify or control for the effects of phylogeny. For example, some studies have explicitly sampled only geographically and phylogenetically distant societies (i.e. the standard cross-cultural sample [81]) to minimize the effects of horizontal and vertical transmission when testing hypotheses about the adaptive or social value of a trait (e.g. [82]). However, this stratified sampling is not completely effective—the standard cross-cultural sample does still have substantial and significant autocorrelation [83], and it is better to tackle the problem head-on with phylogenetic methods rather than hoping that a stratified sample is sufficient [84].

A number of recent approaches have instead actively sought to quantify the relative contributions of ancestry, diffusion and/or environmental selection to a variety of cross-cultural phenomena that are highly variable in their expression (e.g. [9,85,86]). These studies have relied on the analyses of large global datasets, using phylogenetic methods (i.e. phylogenetic regression; figure 1*b*) that take advantage of the fact that neighbouring societies are not always close relatives and/or do not always inhabit similar environments (hence allowing the methods to partial out the relative effects of each driver, e.g. [10,85,86]).

In these studies, the potential for horizontal diffusion has often been assumed to be proportional to either the trait's representation within nearby cultural ‘neighbourhoods' (e.g. [85]) or the geographic proximity to neighbouring groups (e.g. [9]). However, these proxies are likely to be problematic. Specifically, neighbourhood proxies will often suggest a high potential for cultural diffusion if the trait of interest is spatially clustered, regardless of whether diffusion actually existed. Similarly, the use of centroid distances between cultural ranges, as typically defined in phylogenetic–spatial regressions, does not necessarily capture the actual amount of contact between neighbours. This problem arises because the size of cultural ranges tends to increase with latitude [87], meaning that centroid-to-centroid distances are likely to increase with latitude even if border-to-border distances do not. As a result, phylogenetic–spatial regressions based on centroid distances could exhibit systematic underestimation of the potential for cultural diffusion as we move away from the tropics. A further dilemma is how to account for historical migrations/relocations of groups through (pre)history. In using spatial coordinates from one point in time to identify neighbouring cultures, current methods inevitably give precedence to neighbourhood effects from that time. Spatial neighbourhood approaches also do not account for long-distance borrowing among groups that may reflect contact through networks for resource extraction, trade, religion and/or conquest.

#### (ii) Alternative approaches

Possible ways to more accurately disentangle the roles of space, environment, cultural diffusion and phylogeny without abandoning tree-based approaches might include approximating the potential for cultural diffusion by measuring the strength of connections in known contact networks, quantifying actual travelling times between known population centres using historically appropriate means of transportation, and estimating territorial overlap or shared boundaries rather than distances among centroids. However, the difficulties of modelling complex pathways of diffusion in cultural evolutionary studies have led some researchers to abandon altogether the idea that cultural relationships should be approximately tree-like and model them instead as reticulated networks [88].

**Figure 3.**
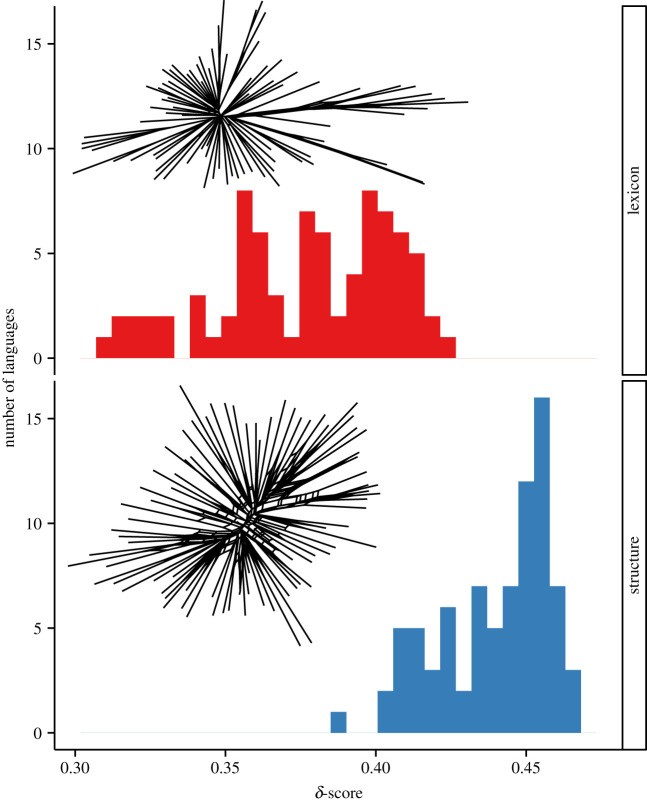
Histogram showing the phylogenetic fit of the lexical and grammatical/structural features of the languages from Greenhill *et al*. [73] as measured by the delta (*δ*)-score [50,92]. The grammatical/structural data show higher *δ*-scores, indicating a far worse fit to the overall phylogeny of Austronesian languages. The networks inset demonstrate the conflicting signal in these data visually, with the lexicon having a more tree-like pattern with fewer conflicts. (Figure is taken from [73] with permission.)

Phylogenetic network approaches were developed for biological entities for which hybridization, horizontal gene transfer and recombination are common. As such, they are also of great interest for the modelling of cultural and linguistic evolution. Unlike phylogenetic trees, phylogenetic networks allow for hybrid nodes (nodes with two parents). Phylogenetic networks can be unrooted, semi-rooted or rooted. Rooted phylogenetic networks, like trees, can provide estimates of the timing of divergence, convergence (hybridization) and horizontal transfer of genetic (cultural) material (e.g. [89]). Note that there is an important distinction between ‘implicit' or ‘data display' networks and ‘explicit' or ‘phylogenetic' networks [90,91]. Data display/implicit networks are increasingly used to graphically represent conflict in phylogenetic trees (e.g. figure 3). However, in these networks, internal nodes do not represent ancestors but noise or conflict in the tree signal. By contrast, internal nodes on explicit phylogenetic networks are meaningful such that hybrid nodes explicitly represent ancestral contact events.

In spite of their potential, explicit phylogenetic network approaches to inferring cultural phylogenies remain rare. In a recent review of their application to genomic data, Blair & Ané [93] summarize three major obstacles to their broader application. First, network inference is computationally intensive. Inference based on ‘full network' comparisons is currently only possible for small numbers of taxa (in the order of 5–10 taxa). Researchers working with larger samples of taxa could, in theory, run the network inference methods on multiple subsets of taxa. However, the researchers would then face the question of how to integrate topologies inferred for each subset, many of which may be unreliable simply for having been inferred from a very small sample (spurious findings of low to no phylogenetic signal are more common when examining trait distributions across a small number of taxa, and topologies inferred from small samples are more susceptible to distortion by ‘long-branch attraction' [93–95]). A second major obstacle is the paucity of methods for selecting the ‘best' network from a series of inferred networks, particularly when some are more complex than others. One solution is to compare only a small number of plausible networks of similar complexity (e.g. [96]). Indeed, methods for identifying cases where network approaches are appropriate, and for selecting a small set of ‘plausible' networks for comparison, have been the focus of recent work in cultural evolution (e.g. [97]). A final obstacle to the adoption of network inference is its greater susceptibility, relative to tree inference, to violations of model assumptions (e.g. assumption of constant rates) which can lead to incorrectly inferred horizontal transmission events [93].

In view of these obstacles, Blair & Ané [93] argue it would be pre-emptive to abandon tree-based inference in favour of networks. Instead, they argue for the continued development of phylogenetic network approaches and in particular of unbiased model selection methods that would allow researchers to weigh the suitability of alternative reticulated topologies. The authors also suggest fruitful paths for combining tree- and network-based inference methods that take advantage of the different strengths of the two approaches (e.g. [98,99]).

### (e) Are model shortcomings giving unwarranted precedence to tree-like inheritance patterns?

The correlated nature of the drivers of cross-cultural similarity creates yet another non-trivial issue for evolutionary analyses that attempt to disentangle them. Specifically, whenever a model ‘corrects' for one of these potential effects, it is implicitly assuming that such effect takes precedence over all others. For example, phylogenetic generalized least-squares (PGLS) regression only tests the effects of potential ecological predictors after discounting cross-cultural similarities already expected from phylogenetic relatedness. In other words, these models implicitly assume that cultural similarity is first explained by descent, which as we know from some well-documented case studies may not always be the case. Take, for example, a trait that diffuses very quickly. Because neighbouring cultures are often close relatives, a PGLS framework might incorrectly attribute observed spatial and phylogenetic clustering to vertical transmission and may lead interested researchers to miss the evidence for cultural diffusion altogether. A potential methodological solution to this problem is to assume that the baseline level of cross-cultural similarity is a joint function of phylogenetic and geographic distance. Recently, implemented spatio-phylogenetic models [100,101] use this approach and can estimate the most likely relative contribution of spatial and phylogenetic processes from the data themselves. Here too, though, it should be noted that the method is implicitly assuming a hierarchy of effects, in which diffusion and vertical transmission take precedence over ecological selection (rendering the burden of proof for an effect of the latter much higher than for the former). Alternatively, when competing mechanisms are well known and amenable to simulation, researchers may use generative inference [102] to identify the most likely evolutionary mechanism. Generative inference involves comparing the patterns and or variable values observed in real data with the distribution of similar parameters obtained from simulations with alternative mechanistic scenarios.

## 5. Conclusion

One of the strongest appeals of cultural tree thinking is that it offers a possible way to illuminate the unobservable past and thus make causal inferences about the processes that have shaped human history. However, throughout this paper, we have cautioned that inferring processes from pattern requires careful consideration and validation. We would stress that cultural phylogenies should be treated as just one tool and one line of evidence. Other lines of evidence should also be explored, and we encourage researchers to consider the potential for multiple evolutionary processes (e.g. diffusion, ecological selection, cognitive constraints and descent), when comparing phylogenetic inferences with what is known from archaeology, anthropology and linguistics. As the field grows, new parallel data will make more robust benchmarking feasible. For this to happen, datasets must be openly accessible and transparent regarding both their primary sources and coding definitions. The use and continued development of statistical methods that measure and disentangle phylogenetic signal from other drivers of cross-cultural diversity are also much needed. With careful use, phylogenetic methods can continue to play a crucial role in uncovering the broad patterns of change in human cultural history and the processes that have shaped them.

## Data accessibility

This article has no additional data.
